# Therapeutic Potential and Pharmaceutical Development of Thymoquinone: A Multitargeted Molecule of Natural Origin

**DOI:** 10.3389/fphar.2017.00656

**Published:** 2017-09-21

**Authors:** Sameer N. Goyal, Chaitali P. Prajapati, Prashant R. Gore, Chandragouda R. Patil, Umesh B. Mahajan, Charu Sharma, Sandhya P. Talla, Shreesh K. Ojha

**Affiliations:** ^1^Department of Pharmacology, R. C. Patel Institute of Pharmaceutical Education and Research, North Maharashtra University Shirpur, India; ^2^SVKM Institute of Pharmacy Dhule, India; ^3^Department of Internal Medicine, College of Medicine and Health Sciences, United Arab Emirates University Al Ain, United Arab Emirates; ^4^Department of Pharmacology and Therapeutics, College of Medicine and Health Sciences, United Arab Emirates University Al Ain, United Arab Emirates

**Keywords:** thymoquinone, pharmacological properties, therapeutic potential, formulation development, safety and adverse effects

## Abstract

Thymoquinone, a monoterpene molecule is chemically known as 2-methyl-5-isopropyl-1, 4-benzoquinone. It is abundantly present in seeds of *Nigella sativa* L. that is popularly known as black cumin or black seed and belongs to the family *Ranunculaceae*. A large number of studies have revealed that thymoquinone is the major active constituent in *N. sativa* oil this constituent is responsible for the majority of the pharmacological properties. The beneficial organoprotective activities of thymoquinone in experimental animal models of different human diseases are attributed to the potent anti-oxidant and anti-inflammatory properties. Thymoquinone has also been shown to alter numerous molecular and signaling pathways in many inflammatory and degenerative diseases including cancer. Thymoquinone has been reported to possess potent lipophilicity and limited bioavailability and exhibits light and heat sensitivity. Altogether, these physiochemical properties encumber the successful formulation for the delivery of drug in oral dosages form and restrict the pharmaceutical development. In recent past, many efforts were undertaken to improve the bioavailability for clinical usage by manipulating the physiochemical parameters. The present review aimed to provide insights regarding the physicochemical characteristics, pharmacokinetics and the methods to promote pharmaceutical development and endorse the clinical usage of TQ in future by overcoming the associated physiochemical obstacles. It also enumerates briefly the pharmacological and molecular targets of thymoquinone as well as the pharmacological properties in various diseases and the underlying molecular mechanism. Though, a convincing number of experimental studies are available but human studies are not available with thymoquinone despite of the long history of use of black cumin in different diseases. Thus, the clinical studies including pharmacokinetic studies and regulatory toxicity studies are required to encourage the clinical development of thymoquinone.

## Introduction

Our nature is surrounded by numerous plants that are used in the welfare of mankind including their use for dietary, culinary, medicinal, and curative purposes. Many of the traditional and alternative medicines rely on the use of medicinal plants for promotion of general health and well-being due to their diverse pharmacological effects. The systematic scientific studies have revealed the power of healing with the use of these plants and herbs due to the active chemicals known as phytochemicals that belongs to many classes such as saponins, alkaloids, terpenes, and glycosides present in the herb. These compounds possess different moieties which provide pharmacophore for drug development. Among different chemical moieties, quinone is one of the most abundant groups including naphthoquinone, anthraquinones, and benzoquinone. From the family of these quinones, benzoquinone that is represented by thymoquinone (TQ) has received enormous attention for its pharmacological properties and therapeutic potential (Darakhshan et al., 2015).

TQ is the most abundant bioactive component of volatile oil of *Nigella sativa L*., a member of *Ranunculaceae* family. The seeds of *N. sativa* are faithfully used for dietary purposes in Middle East countries and popularly known as black cumin. It was reported that the biological activities of *N. sativa* seeds are mainly ascribed to its essential oil constituent that is TQ (30–48%) and was first extracted by El–Dakhakhny (1963). The black seed oil is cataloged in the list of United States Food and Drug Administration as “Generally Recognized as Safe.” The major pharmacological activities exerted by TQ included anti-convulsant, anti-microbial, anti-cancer, anti-histaminic, anti-diabetic, anti-inflammatory, and anti-oxidant. It has been found to elicit potent anti-oxidant activity due to the potent free radical scavenging action against superoxide anions and raising the transcription gene responsible for the production of natural anti-oxidant such as superoxide dismutase (SOD), catalase (CAT), and glutathione peroxidase (GSH; Ismail et al., 2010).

The pharmaceutical development of TQ becomes a crucial assignment and brings challenges in the drug discovery and development. TQ bears potent hydrophobicity or lipophilicity nature that is well-evidenced by the value of log *P* = 2.54. This demonstrates that hindrance in the pharmaceutical development of TQ to formulate it into the conventional dosage forms such as tablet and capsule. Further, the formulation aspects were also hindered due to its highly thermolabile nature. Therefore, numerous strategies for the formulation of TQ have been developed in the recent past including the fabrication of TQ using the novel nanoformulations. These novel strategies may overcome the barrier in pharmaceutical development and improve the bioavailability of TQ without compromising the efficacy and safety. In the present article, we reviewed the sources, major pharmacological targets, molecular mechanism underlying the pharmacological effects. The drug delivery approaches including the nanotechnology to overcome the bioavailability and target related obstacle of TQ are also reviewed herein.

## Search methodology

Database using Google scholar, PubMed, and Scopus internet search engines were utilized for the literature search updated documented information regarding thymoquinone up to 31st March 2016. The literature search was restricted to language English only. For data extraction and retrieval, following key words were used in the database mentioned above. The Boolean operator words such as AND/OR was used between the words to retrieve maximum literature. The keywords were thymoquinone LD50, thymoquinone in cancer, sources of thymoquinone, extraction process of thymoquinone, pharmacokinetics of thymoquinone, analogs of thymoquinone, thymoquinone, and cancer targets, thymoquinone formulations, thymoquinone in cardiac arrest, thymoquinone organ protective agent, thymoquinone PPAR, thymoquinone oxidative stress, thymoquinone hepatoprotection, thymoquinone tumor proliferation, thymoquinone anti-inflammatory, thymoquinone hypertension, thymoquinone anti-microbial, thymoquinone brain, thymoquinone neuropathy pain, thymoquinone gastroenterological, thymoquinone kidney, thymoquinone renal, thymoquinone heart, thymoquinone toxicity, thymoquinone clinical trial, thymoquinone carbon nanotubes, thymoquinone liposomes, thymoquinone dendrimers, thymoquinone Nano emulsion, thymoquinone polymeric micelle, thymoquinone niosome, thymoquinone solid-lipid nanoparticles etc. Nearly all the associated and cross reference articles were screened and pertinent data was extracted.

## Sources of thymoquinone

*N. sativa* whose seeds commonly known as black cumin are the main natural source of TQ (Havlik et al., 2006). Apart from *Ranunculaceae* family, the presence of TQ in several other genera of the *Lamiaceae* family including as *Agastache, Coridothymus, Monarda, Mosla, Origanum, Satureja*, and *Thymus* were also reported (Hirobe et al., 1998; Economakis et al., 2002; Ipek et al., 2005; Lukas et al., 2009). It has also been found in genus *Tetraclinis*, and in the form of glycoside in the genera *Cupressus* and *Juniperus* of the *Cupressaceae* family. The traces of TQ were also found in *Nigella arvensis* L. seeds (Havlik et al., 2006)., TQ in many plant species is present in dimeric and reduced forms such as dithymoquinone (DTQ) and thymohydroquinone (TQ), the second being considered as a compound with potential pharmacological activities including anti-bacterial (Toama et al., 1974), anti-fungal (Halamova et al., 2010), anti-inflammatory, anti-oxidant, and acetyl cholinesterase inhibitory (Jukic et al., 2007). The presence of TQ in various parts of plants along with their family and species are provided in Table 1.

**Table 1:** The content of thymoquinone in different plants.

**S. No**	**Family**	**Species**	**Part of plant**	**Content (mg/kg) TQ**	**References**
1	Asteraceae	*Eupatorium cannabinum*	Aerial	8	Toama et al., 1974
2	Cupressaceae	*Juniperus communis* L.	Twig	6, 15	Toama et al., 1974
3	Lamiaceae	*Monarda didyma* (chemotype 1) *Monarda didyma* (chemotype 2) *Monarda didyma* L. pink lace Monarda media. wild Monarda menthifolia. *urejamontana L.* *Satureja hortensis* L. *Thymus pilegioides* L. *Thymus serpyllum* L. *Thymus vulgaris*	Aerial Inflorescence Leaf Stem Aerial Aerial Aerial Aerial Aerial Aerial Aerial Aerial	3,029 3,564 821 23 670 2,995 1,381 1,052 217 223 233 300	Hirobe et al., 1998; Economakis et al., 2002; Ipek et al., 2005; Lukas et al., 2009
4	Ranunculaceae	*Nigella sativa* L.	Seed	1,881	Havlik et al., 2006

## Extraction process of thymoquinone

TQ is extracted from essential oil obtained from the seeds using various methods which are outlined in next paragraphs.

### Supercritical fluid extraction (SFE)

The supercritical fluid extraction was carried out using food-grade liquid CO_2_ in high-pressure cylinders. The CO_2_ was drove into the SFE system till the required pressure of CO_2_ was attained. The pressure regulators were used to achieve the perfect pressure regulation. The SFC vessel was filled with the 1 kg of dried powder of cumin seeds. The heat exchangers were used to set the temperature of the system. The supercritical CO_2_ flows from the extractor the then goes to the separator vessel. The samples from the extractor were collected and flow of the CO_2_ was maintained by flow meter. The complete extraction were carried out in two different environment by changing the pressure and temperature (Salea et al., 2013).

#### Hydrodistillation

The supercritical fluid extraction using solid CO_2_ extract was used for the hydro-distillation (HD) for 6 h using a Clevenger-type apparatus. The yellow color essential oil (HD-SFE) with aromatic odor was obtained. The yield was dried by anhydrous sodium sulfate (Al-Haj et al., 2008).

#### Soxhalation

Initially, the air-dried plant material was homogenized using a laboratory mill. According to the report, 5 g of dried powder was weighed into a paper cartridge and extraction was carried out 6 h in the Soxhlet apparatus. Subsequently, the extract was filtered using anhydrous sodium sulfate and re-extracted three times with methanol (30 ml) in a separator funnel. Lastly, the methanol portions were collected in a round bottom flask and methanol was removed in a vacuum rotary evaporator and 10 ml of the internal standard solution (BHT 1 mg/ml) was added (Salman et al., 2016). The different quantification techniques for TQ is given in Table 2.

**Table 2:** The quantification techniques used for TQ in different samples.

**Mobile phase used**	**Extraction of sample**
Methanol and 10 mM KH_2_PO_4_ buffer in the ratio of 90:10	Methanol + acetonitrile
Methanol: water (92:8)	Methanol, 20 min vortex
Water:methanol:2propanol (50: 45:5 %v/v)	–
Water: ACN (45:55% v/v)	Ethyl acetate

## Development of analogs of thymoquinone

Numerous studies have been involved to develop the analogs of TQ with remarkable efficacy for different types of cancers. Recently, the novel analogs of TQ were analyzed with various monoterpenes, sesquiterepene, and cytotoxic terpenes for better anti-cancer activity. These analogs were tested against various cancer cell lines to demonstrate cell specific activity. The resulting data proposed that some of these analogs were more potent than TQ, the parent drug at the same time being less cytotoxic to the non-malignant cells.

## Pharmacokinetics of thymoquinone

In animal studies, different dose regime of TQ which were administered either by intraperitoneal or intravenous or intragastric routes were studied for their efficacy in diseases models. The experimental studies in animals reveal the usual tested therapeutic doses of TQ 5 mg/kg (intravenous) and 20 mg/kg (per oral; Alkharfy et al., 2015). The pharmacokinetic parameters were calculated using high performance chromatography (HPLC) in the collected blood samples from vole rabbits. The mobile phase contains methanol and potassium dihydrogen phosphate buffer and carried at the rate of 0.9 ml/min. The compound, TQ was detected at the wavelength of 254 nm. The estimated clearance (CL) following IV administration was found to be 7.19 ± 0.83 ml/kg/min, and volume of distribution at steady state (Vss) was 700.90±55.01 ml/kg. While, subsequent oral dosing, the CL/F and Vss/F-value were found to be 12.30 ± 0.30 ml/min/kg and 5,109.46 ± 196.08 ml/kg, respectively. These parameters remained connected through elimination half-life (t_1/2_) of 63.43 ± 10.69 and 274.61 ± 8.48 min with intravenous and oral administration. The proposed absorption t_1/2_ was about 217 min. The compartmental analysis revealed t_1/2a_ of ^*^8.9 min and t_1/2b_ of ^*^86.6 min. The projected complete bioavailability of TQ stayed ^*^58 % through a lag time of ^*^23 min (Alkharfy et al., 2015). The protein binding of TQ was found 99% with a composite rapid abolition and moderately gentler absorption following oral administration. Taken together, the data demonstrate that TQ has enormous potential for its clinical usage and pharmaceutical development. Though, the solubility and bioavailability is a major hindrance in drug discovery and development. So, many new strategies of targeted drug delivery and newer techniques were developed including nanotechnology.

## Physiochemical and pharmaceutical properties of characteristics

### Solubility studies

The solubility is often signified as the characteristic property of drug to mentor the use of the drug. The relevant literature showed the solubility of TQ in commonly used solvents such as water, 0.1 N HCl and PBS (at pH 5, 7.4, and 9) as this data are essential for the successful formulation. It was seen that solubility of TQ remains the same in almost all the above solvents.

### Stability studies

The stability of TQ was evaluated using HPLC. It was observed that TQ was more stable at lower pH and stability decreases if increase in pH eventually. The degradation rate of TQ at lower pH was minimal, while it goes on increasing as the pH turns basic.

### Effect of light

A data in relevant literature reveals that even for short period of exposure TQ showed higher sensitivity. The kinetics of photolysis of TQ was studied using ethanol. The outcomes showed a fast deprivation of TQ (more than 70%) in leading 10 hrs and the procedure remained detected in second order kinetics. From the above data it was observed that the solubility of TQ was compromised by the stability of TQ. It was also revealed that TQ is highly unstable in aqueous medium at prominent light and pH. Hence, for *in-vivo* administration of TQ, it is advisable to use oil phase to solubilize TQ and to overcome the stability issues related to the TQ.

### Quantitation of thymoquinone

HPLC has been routinely used in bioanalytical studies to detect the traces of TQ in plasma and serum. The relevant studies suggest many mobile phases used to quantify the traces of TQ. The documented methods have been mentioned in Table 3.

**Table 3:** The anti-cancer activity of thymoquinone in different cancer cell lines.

**Cancer**	**Cell lines**	**Action**
Fibrosarcoma and Leukemia	p-53 deficient acute lymphoblastic leukemia Jurkat cell line	↑ p73 expression, ↑ UHRF1 expression
Pancreatic and Gastric cancer	FG/COLO357 and CD18/HPAF	↓ NF-kB, ↓ MUC4, ↑ p-38 ↓ bcl-2, ↑ bax, ↑ caspase-6 and -9
Breast and Lung cancer	NCI-H460, NCI-H146, MCF-7/DOX T47D	↑ PPAR- γ, ↓ bcl-xL, ↓ survivin, ↑ PTEN mRNA. ↑ Bax/Bcl2 ratio, ↑ Bax,↓ Bcl2, ↓ ENA-78 and ↓Groalpha, ↓ NF-kB
Prostate and Colorectal cancer	LNCaP, C4-B, DU145, and PC-3 colon cancer cells HCT116	↓ Androgen Receptor ↓ E2F, ↑ CHEK1, ↑ p53

## Molecular mechanisms of thymoquinone

### Effect on inflammatory mediators

Cyclooxygenase (COX) also recognized as prostaglandin (PG) endoperoxide synthase catalyses the phases of blend of prostanoids (Minghetti, 2004). COX1 and COX2 are the two types of COX enzymes. COX1 is regulated by the growth factor and cytokines and its expression in almost all tissues is in inducible isoform, which is overexpressed in inflammatory conditions (Ramsay et al., 2003). While, overexpression of COX2 was found in a wide range of cancers such as lungs, stomach, breast, and pancreatic cancer (Khuri et al., 2001; Singh et al., 2005; Cascinu et al., 2007; Kaseb et al., 2007; Richardsen et al., 2010). The overexpression of COX2 plays a crucial role as prostaglandin up-regulates angiogenesis and increases the resistance to apoptosis (Costa et al., 2002; Santos and Costa-Pereira, 2011). COX2 is reported for the inhibition of some clinical behavior of tumors (Banerjee et al., 2009; Yu and Kim, 2012). The inhibitor of COX2 shows a significant effect on its inhibition, while it also possess some side effect on other tissues, therefore in recent years natural product received attention for its choice to prevent cancer through the COX2 inhibitory mechanism.

TQ has revealed a substantial data on the reticence of COX2 expression and PG production in allergic airway inflammation in mice (El Mezayen et al., 2006). TQ attenuated the FLMP induced inflammation by impairment of phosphorylation on Ser-304 and Ser-328 of p47*PHOX* phosphor peptides. TQ also revealed the protection in FMLP stimulated polymorphonuclear cell by declining the expression of gp91PHOX and CD11b and inhibition of myeloperoxidase (Boudiaf et al., 2016).

### Effect on peroxisome proliferator-activated receptors (PPARs)

PPARs are ligand-activated transcription factors that plays a vital role in tumor suppression by inducing apoptosis and exerting anti-proliferative actions in various cancer cell lines (Kubota et al., 1998; Sarraf et al., 1998; Chang and Szabo, 2000; Yin et al., 2001; Kumar et al., 2009; Venkatachalam et al., 2009; Alhosin et al., 2012). The ligands of PPARs showed potential in the prevention of cancer by regulating various genetic pathways via activation and up-regulation of PPARs. TQ showed upregulation of PPAR-γ and downregulation of the genes such as Bcl-2, Bcl-xL involved in cell death and survival mechanism in the breast cancer cells (Mangelsdorf et al., 1995; Woo et al., 2011). Further, in molecular docking studies, TQ appears to interact with 7 polar residues and 6 non-polar residues in the PPAR-γ (Feige et al., 2006; Woo et al., 2011). TQ was also observed to induce the activation of other isoforms of PPARs including such as PPAR-β/δ in breast cancer cells. The findings demonstrated PPAR-γ activation mediated anti-cancer activity of TQ. The role of PPAR-γ activation was further confirmed by abrogation of the TQ incited apoptosis and reduced survivin in MCF-7 cells by GW9662, a well-known PPAR-γ inhibitor (Minghetti, 2004; Hussain et al., 2011). Additionally, apart from anti-cancer activity which is a new experimental therapeutic target for cancer, the PPAR-γ agonist activity could be important in providing a pharmacological basis of its use in diabetes, wherein PPAR-γ agonists are clinically used for the management.

### Effect on oxidative markers

Aerobic metabolism as well as UV radiation and environmental pollution causes the persistent generation of superoxide anion radical (O2−^•^), hydroxyl radical (^•^OH) and peroxyl radical (ROO^•^). The sustained elevated levels of ROS cause oxidation of biomolecules including lipid membrane, protein and nucleic acids. TQ possesses free radical scavenging activities against free radicals which initiate oxidative stress in various *in vitro* and *in vivo* animal models. Due to potent anti-oxidant and free radical scavenging action, TQ has been shown to normalize the adverse effect of various environmental toxins or xenobiotics causing oxidative damage and organ dysfunctions and leads pathogenesis of various diseases (Mansour et al., 2001; Kanter, 2008; Alkharfy et al., 2013). TQ also impede the generation of oxidative stress by countering xanthine/xanthine oxidase system (Badary et al., 2000). SOD shows a vital role in altering superoxide anions into hydrogen peroxide and oxygen; hence it forms the first line anti-oxidant defense and known as an important detoxification enzyme. TQ has been shown to enhance the activity of SOD. Glycation of SOD upon *in vitro* incubation of the SOD with glucose or methylglyoxal results in decreased activity of SOD that is an additional mechanism of TQ in modulating SOD activity and subsequent retention of the anti-oxidant activity (Khan et al., 2014). TQ also showed the scavenging of the carbon-centered radicals and hydroxyl radicals as assessed in the *in vitro* assays; 1,1-diphenyl-2-picrylhydrazyl (DPPH) and iron-catalyzed injury of deoxyribose. Further, it also curtailed the development of oxidative stress by non-enzymatic interface involving the glutathione (GSH) cycle to produce glutathionylated-dihydrothymoquinone (Khalife and Lupidi, 2007). Both the reduced metabolites of TQ exhibited strong free radical hunting action against 2,2-azinobis (3-ethylbenzothiazoline-6-sulfonic acid) and DPPH assay by giving hydrogen to free radicals. Since lipid peroxidation products form adduct through proteins or DNA, the inhibitory effect of TQ on lipid peroxidation in part accounted for its beneficial effects. Altogether, the studies demonstrate that TQ attenuate cellular oxidative stress by inducing GSH under different experimental pathologic conditions (Badary et al., 1997; Badary, 1999; Fouda et al., 2008). Convincing data demonstrate that the uncontrolled generation of free radicals target the lipid membranes and induce a state of anti-oxidant defense that leads the initiation of lipid peroxidation. The occurrence of lipid peroxidation is an important pathogenic event in many diseases and can be measured by the biomarkers of lipid peroxidation *in vitro* and *in vivo* models of human diseases. Until now, convincing number of experimental studies have recommended that TQ provide protection against many chemicals and drugs such as ifosfamide, mercuric chloride, cyclophosphamide, isoproterenol, cisplatin, and doxorubicin by augmenting anti-oxidant anti-oxidant defense and attenuating inflammation and apoptosis (Badary et al., 1997; Badary, 1999; Fouda et al., 2008). TQ not only confer prevention against the pathologic changes in different disease, but also reduced the adverse effects of major drugs used in chemotherapy and enhances the susceptibility of tumors for the chemotherapeutic agents and appear as chemopreventive as well as chemosensitizers and organ protective. The suppression of DMH-induced rat colon carcinogenesis through TQ has been shown to be associated with reduced generation of ROS and development of conjugated accumulation of malondialdehyde (MDA; Jrah-Harzallah et al., 2013; Lang et al., 2013). TQ also ameliorated carbon tetrachloride prompted hepatotoxicity as demonstrated by decrease in the raised levels of serum enzymes and refurbishment of GSH in liver (Nagi and Mansour, 2000; Mansour et al., 2002). TQ augmented GSH toward normal and repressed *in vitro* fabrication of superoxide anions, thus proposing defense in N (omega)-nitro-l-arginine methyl esters (L-NAME) persuaded injury via anti-oxidant possessions (Khattab and Nagi, 2007).

**Figure 1:**
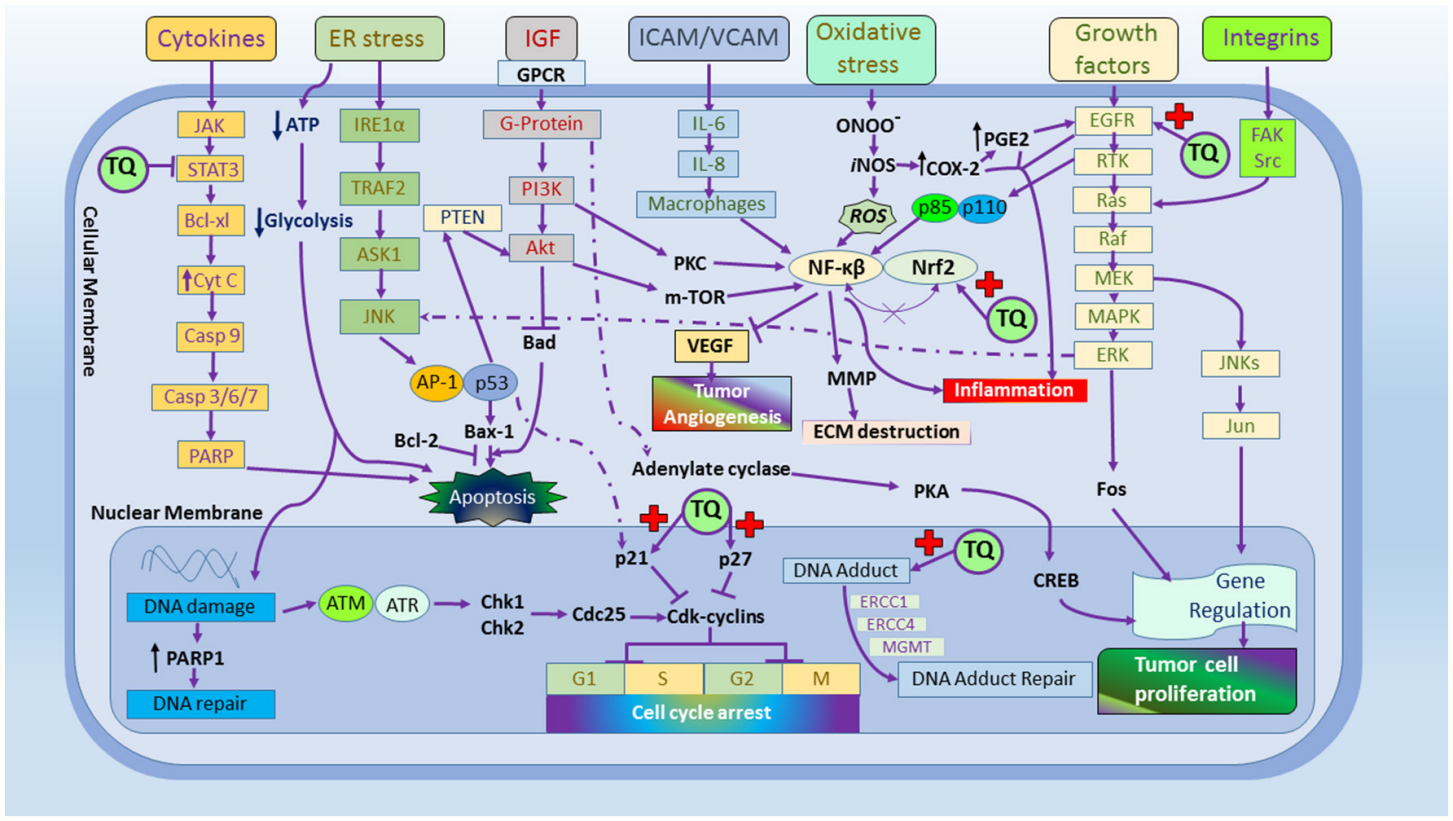
The molecular targets of thymoquinone.

Nagi et al. showed that TQ may act as a substrate for mice hepatic DT-diaphorase in the occurrence of abridged arrangement of nicotinamide adenine dinucleotide (NADH) where, TQ found to decrease dihydrothymoquinone (DHTQ), which is more effective than TQ and butylated hydroxytoluene (BHT; Nagi and Almakki, 2009). Al-Shabanah et al. found TQ effective in culminating GSH transferase along with quinone reductase (QR) in mouse liver and indicated TQ an important prophylactic agent against chemical carcinogenesis and toxicity (Al-Shabanah et al., 1998). Badary and Gamal showed the inhibitory potential of TQ in a fibro sarcoma animal model associated with decrease in the hepatic lipid peroxides and amplified GSH and enzyme activities of GST and QR, demonstrating its potential as chemopreventive and/or therapeutic agent (Badary and Gamal, 2000). Different mechanisms were highlighted in Figure 1.

### Effect on apoptosis

Imbalance in tumor suppressor genes leads to the development of cancer as the inhibitor actions are subsided. TQ plays a vital role in chemoprevention by activating tumor suppressor genes. Phosphatidylinositol-3,4,5-trisphosphate (PIP3), which is a major substrate of PTEN a multifunctional phosphatase (Maehama and Dixon, 1998) and the lipid phosphatase activity of PTEN play a crucial role in dephosphorylation of PIP3 and also inhibit the Akt/PI3K pathways (Rahmani et al., 2012). However, PI3K/Akt inactivation and up-regulation of PTEN are of vital importance in chemoprevention. An important study showed time dependent upsurge of PTEN in MCF-7/DOX cells and it was observed that TQ treatment caused an rise in PTEN mRNA by 1.8-, 2.0-, 3.8-, 5.9-, and 7.9-fold after 1, 2, 4, 8, and 24 h, correspondingly (Arafa et al., 2011; Liu et al., 2016). TQ also plays a significant role in the modulation of other tumor suppressor genes such as p53 (altered in ~50% of cancers), p21, and p27, confirming its anti-apoptotic activity. TQ as well as a standard anti-cancer drug, 5-FU both were found to decrease Bcl-2 and increase Bax and the release of cyt-c from the mitochondria. TQ and 5-FU both have induced apoptotic cell death by inducing caspase-3 and caspase-9 initiation in stomach tumor cells (Lei et al., 2012; Paramasivam et al., 2012). TQ treatment modulated Src homology-2 phosphatase 2 concomitant to the diminution of STAT3 activation. TQ treatment also down-regulated STAT3-regulated genes for example cyclin D1, Bcl-2, Bcl-xL, survivin, Mcl-1, and vascular endothelial growth factor. Further, TQ arrested cell cycle in G1 phase and induced apoptosis as demonstrated by poly-ADP ribose polymerase cleavage (PARP; Li et al., 2010). TQ also induced time-dependent proteasome complex both in 20S and 26S proteasomes and in U87 MG and T98G glioblastoma cells that elucidate the mechanistic induction of apoptosis in cancer cells (Cecarini et al., 2010; Kolli-Bouhafs et al., 2012). TQ treatment also modulated apoptosis by DNA disintegration, stimulation of caspases and cleavage of PARP as well as increased the Bax/Bcl-2 ratio in A431 and Hep2 cells (Das et al., 2012). Further, the apoptosis caused by TQ was shown associated with impaired mitochondrial potential and induction of caspases. Furthermore, TQ treatment augmented Bax/Bcl2 ratio *via* up-regulating Bax and down-regulating Bcl-2 proteins. Additionally, PTEN silencing by target specific siRNA enhanced inhibition of TQ-induced apoptosis that results in improved cell resistance (Arafa et al., 2011). The G1 phase arrest up to 24 h with TQ treatment showed prolonged phase shift to sub G1, representing apoptosis as confirmed by loss of cyclin D1, cyclin E, and cyclin dependent on kinase inhibitor p27. Immunoblot and mitochondrial potential studies showed mitochondrial impairment and apoptosis with up-regulation of Bax, cytoplasmic cytochrome c and procaspase-3, PARP cleavage through Bcl-2, Bcl-xL. Rajput et al. envisaged mechanistic insight on mitochondrial dysfunction by exploring the phosphorylation of PDK1, PTEN, Akt, c-raf, GSK-3β, and Bad in TQ preserved cells and showed the participation of Akt in apoptosis. Consequently, TQ-induced Akt reserve was proven by translational domination through phosphorylation of 4E-BP1, eIF4E, S6R, and p70S6K (Rajput et al., 2013).

TQ was observed to induce apoptosis in the human colon cancer cells and animal models through p53 dependent pathway (Gali-Muhtasib et al., 2004, 2008) which caused 2.5- to 4.5-fold surge in mRNA appearance of p53 and the decrease in p53 target gene, p21WAF1 (Gartel et al., 1996; Gartel and Tyner, 1998). Also, another study showed that TQ induces apoptosis via up-regulation of PTEN at transcription level in doxorubicin-resistant breast cancer cells. Additionally, the up-regulated PTEN modulated the p53 and p21 protein expression mediating PI3K/Akt pathway. In p53-null myeloplastic leukemia in HL- 60 cells, TQ-induced apoptosis by activating caspase-3, caspase-8 and caspase-9 (Arafa et al., 2011). Overall, the data suggest that TQ inhibited the tumor by up-regulating the tumor suppresser genes such as PTEN and by supressing the Akt/PI3K pathways which is suggestive of its novel therapeutic target and mechanism in cancer.

### Effect on transcription factors

Nrf-2 is a cell-signaling transcript factor that marks cytoprotective phase II anti-oxidant enzymes such as GST, SOD, CAT, NQO-1, and HO-1. These enzymes detoxify harmful materials in the body and play imperative part in organotropic and chemopreventive effects against anti-cancer drugs like doxorubicin, cisplatin, and cyclophosphamide. TQ is reported to show organ protective effect in cyclophosphamide induced hemorrhagic cystitis model in mice by up-regulation of Nrf-2 factor. NF-kβ expression can be induced by an extensive diversity of stimuli, counting stress, bacteria, viruses, cytokines, and free radicals. This transcript factors regulates the countenance of numerous genes, with enzymes, cytokines, cell cycle regulatory bits, and angiogenic factors (Kanter, 2008). TQ might prevent TNF-α induced NF-kβ activation in HS766T pancreatic ductal adenocarcinoma cells, as well as conquer the translocation of this transcription influence hooked on the nucleus (Chehl et al., 2009).

This was supported by another study that showed TQ suppresses inflammatory stimuli-induced activation of NF-kβ, production of TNF-α, and carcinogens (El-Saleh et al., 2004). This suppression was caused by the inhibition of IkBa degradation and phosphorylation following nuclear translocation. The hang-up of the translocation of NF-kβ into the nucleus in LPS-stimulated macrophages by TQ was additional presented by Wilkins et al. (2010). Though, a study by El Gazzar et al. (2007) have exposed that TQ did not change the translocation of p65 into the nucleus nonetheless, induced the oppressive NF-kβ homodimer requisite to the organizer in LPS-induced RBL-2H3 rat basophil cells (El Gazzar et al., 2007). The favorable properties ensuing after the favorable inflection of NF-kβ by TQ have also been studied in several inflammatory disease models. It has been shown that encephalomyelitis can be ameliorated by TQ possibly through the inhibition of NF-kβ in the rat model of multiple sclerosis (Mohamed et al., 2004). A latest study has established the defensive properties of TQ against rheumatoid arthritis through the reticence of LPS-induced NF-kββ-p65, p38, and ERK1/2 phosphorylation (El-Najjar et al., 2010; Vaillancourt et al., 2011). Therefore, suppression of NF-kβ by TQ is believed to play an important role in its anti-inflammatory activity.

TQ has also been showed to modify the signal transducers and activators (STAT) family of transcription factors (Bromberg and Darnell, 2000). Among, members of the STAT family, STAT3 play an important role in the guideline of transcription of genes intricate in cell differentiation, propagation, apoptosis, angiogenesis, metastasis, and immune reactions (Lupidi et al., 2010). STAT3 is frequently constitutively triggered in numerous kinds of cancers and limits at dissimilar levels of tumorigenesis (El-Najjar et al., 2011). Thus, pointing the STAT3 gesturing trail has been projected as an approach to overturn dissimilar malignancies. Numerous studies have shown that TQ suppress STAT-3 phosphorylation and expression of its downstream signaling effectors Bcl-2, Bcl-XL, cyclin D1, survivin, Mcl-1, and VEGF (Li et al., 2010; Badr et al., 2011). TQ also found to impede constitutive and IL-6-inducible STAT3 phosphorylation in U266 multiple myeloma cells, as well as inhibit c-Src and JAK-2 activation (Badary et al., 1999). STAT3 gene-deleted mouse embryonic fibroblasts that lack activation of STAT3 were found to be more resistant to TQ-induced apoptosis than wild-type fibroblasts, signifying that STAT3 may play a remarkable role in the apoptotic effect of TQ. The same study also reported that TQ down-regulated the expression of STAT3-regulated genes, including cyclin D1, Bcl-2, Bcl-xL, survivin, Mcl-1, and VEGF in U266 cells (Badary et al., 1999). Taken together, these results indicate that ST AT3 is an important target of TQ; though added studies in the *in vivo* models are required to translate the *in vitro* findings *in vivo* and establish the STAT3 based mechanism in attributing the potential effects of TQ.

## Pharmacological potentials of thymoquinone

### Cardioprotective action of TQ

Ojha et al. (2015) demonstrated the cardioprotective effects of TQ in isoproterenol-induced myocardial injury in rats. Isoproterenol is known to induce myocardial lesions similar to acute myocardial infarction in rats. TQ (20 mg/kg) treatment augmented anti-oxidant s and salvaged cardiomyocytes evidenced by restoration of cardiomyocyte injury enzymes and reduction of lipid peroxidation product and pro-inflammatory cytokines. The findings were further supported by histopathological preservation of the myocardium as evidenced by reduced myonecrosis, edema, and infiltration of inflammatory cells. The findings reveled that TQ exert protective effect on cardiac injury by attenuating oxidative stress, enhancing endogenous anti-oxidant s and maintaining structural integrity (Ojha et al., 2015). Attia et al. reported the hypolipidemic possessions of TQ that is arbitrated by free radical scavenging as anti-oxidant activity and reduced hepatic steatosis in hyperlipidemic rabbits. TQ supplementation (20 mg/kg/day) to high cholesterol fed rabbits showed a positive effect on the serum glucose, insulin, and aminotransferases thereby confirming the reduction of reactive oxygen species in fatty liver disease caused by high cholesterol diets fed rabbits. A previous study showed beneficial effects of the *propolis* and TQ on atherosclerosis in cholesterol fed rabbits. The enhanced serum total cholesterol, triglycerides lipoprotein-cholesterol thiobarbituric acid-reactive substances and decreased HDL-cholesterol were ameliorated by both *propolis* and TQ along with beneficial effects on kidney function (Attia et al., 2010; Nader et al., 2010).

Previous reports showed hypocholesterolemic effect of TQ rich fraction (TQRF) from *N. sativa* seeds in comparison with TQ in male Sprague–Dawley rats over 8 weeks. A significant reduction in the plasma total cholesterol levels and low density lipoprotein cholesterol (LDL-C) was observed in the TQRF and TQ treated rats. TQRF and TQ treatment improved the mRNA levels of low density lipoprotein receptor (LDL-R) and 3-hydroxy-3-methylglutaryl- coenzyme A reductase (HMG-COAR). This study demonstrates that TQRF and TQ by diminish rise in cholesterol by modulating the uptake of LDL-C directed LDL-R gene and capsizing the HMG-COAR gene (Nader et al., 2010).

### Anti-microbial action of thymoquinone

Kouidhi et al. demonstrated the anti-microbial action of TQ, tetracycline and benzalkonium chloride using the efflux assay of 4,6-diamidino-2- phenylindole (DAPI) by determining DAPI cell accumulation. TQ provokes a 4-fold potentiation of the verified anti-biotics and anti-septics. TQ also reserved DAPI efflux action evidenced by increased accumulation in clinical isolates and decreased loss from bacteria that indicates the resistance-modifying action of TQ on oral bacteria. Additionally, the anti-microbial activity of *N. sativa* against *S. mitis, S. mutans, S. constellatus*, and *G. haemolysins* were also demonstrated following the administration of essential oil containing 3.35 μg of TQ. The minimum inhibitory concentration of TQ was found to be 19.25 ± 1.6 mg/ml. The study also revealed that TQ exerted potent inhibitory effect on Hep-2 cells as compared to the essential oil delineating its anti-cariogenic activity against bacterial infections (Harzallah et al., 2011; Kouidhi et al., 2011). Further, TQ was also found to prevent bacterial biofilm formation by Chaieb et al. a biofilm is described as the community of cells attached to a biotic or abiotic surface. Minimum biofilm inhibition concentration observed in crystal violet assay revealed that TQ exerted anti-oxidant action mediated anti-microbial activity. The study was carried out on 11 human pathogenic bacteria such as Gram positive cocci *Staphylococcus aureus* ATCC25923 (22 μg/ml) and *Staphylococcus epidermidis* CIP106510 (60 μg/ml; Chaieb et al., 2011). In another study, retaining a sequence of trials, TQ was vigorous in contradiction of *Escherichia coli, Pseudomonas aeruginosa, Shigella flexneri, Salmonella typhimurium, Salmonella enteritidis*, and *S. aureus*. The concentration of TQ prerequisite to constrain and slay *S. aureus* was 400 and 800 μg/ml. The studies suggest that use of TQ and dihydrothymoquinone with currently available modern anti-biotics such as ampicillin, cephalexin, chloramphenicol, tetracycline, and gentamicin may provide synergistic activity in against *S. aureus* (Halawani, 2009).

The anti-fungal activity of TQ has also been studied on eight species of dermatophytes. The anti-fungal activities of *N. sativa* oil, TQ, and griseofulvin were compared and it was found that the oil and its component, TQ showed greater minimum inhibitory concentration than griseofulvin against these fungi (Aljabre et al., 2005). The anti-rhinosinusitis potential of TQ was showed against rhinosinusitis induced by intranasal administration of platelet-activating factor TQ showed promising potential in the treatment of rhinosinusitis and found comparable to the standard anti-biotics. The results were further supported by the histopathological observations (Cingi et al., 2011). In additional to anti-bacterial and anti-fungal activities, TQ was also found effective against *Entamoeba histolytica* and *Giardia lamblia*, the common parasites causing amoebiasis and giardiasis (Sheikh et al., 2015). TQ administered with curcumin shows anti-microbial action by elevating anti-body titer against H9N2 AIV in turkeys. The elevated cytokine gene countenance proposes anti-viral activity of TQ, curcumin and the combination synergistically repressed the pathogenesis of H9N2 viruses (Umar et al., 2016).

### Gastro protective effects of thymoquinone

The efficacy of *N. sativa* oil in treating acute gastric ulcer rat models was demonstrated by Arsalan et al. The authors noted a decrease in lipid peroxidation product; MDA and improved activity of SOD and promoted the recovery of glutathione content was achieved by TQ (20 mg/Kg) in ethanol induced gastric damage and proposed that the effect could be due to the anti-oxidant property of TQ (Arslan et al., 2005). Nagi and Almakki displayed the defensive action of TQ in mice liver that is interceded by the initiation of detoxifying enzymes with quinone reductase (QR) and glutathione transferase (GT). TQ (1, 2, and 4 mg/kg/day p.o) administration for 5 days produced a marked upsurge in the action of QR (147, 196, and 197% of control, correspondingly) and GT (125, 152, and 154% of control, respectively). The other advantage was that TQ showed these protective effects upon oral administration which makes it a promising agent against chemical toxicity (Nagi and Almakki, 2009). D-galactosamine induced liver injury and altered hepatic function was used to demonstrate the hepatoprotective effect of TQ in rats. TQ pre-treatment produced a marked reduction in elevated serum enzymes and hepatic malonaldehyde levels in part mediated by its potent anti-oxidant action. The hepatoprotective activity was found comparable to silymarin, a standard natural hepatoprotective agent. Moreover, the histopathological studies further confirmed the protective effects of TQ as shown by salvage of the liver tissues and its lobular architecture. The findings demonstrate that the membrane stabilizing, anti-oxidant, and free radical scavenging action of TQ in protecting from liver enzyme leakage and lipid peroxidation (Daba and Abdel-Rahman, 1998; Lebda et al., 2011).

Mansour et al. established the properties of orally administered TQ on oxidative stress, lipid peroxidation and DT-diaphorase activity in liver, heart and kidney tissues of mice. TQ given for 5 successive days at the dose of 25, 50, and 100 mg/kg/day produced significant increase in hepatic SOD, CAT, GSHPx, while cardiac SOD activity and lipid peroxidation were decreased at higher doses (50 and 100 mg/kg/day). While, higher dose of TQ an increase in DT-diaphorase activity was observed in cardiac and renal tissues. These reports were reinforced by the synergistic activity of TQ and its metabolite DHTQ (dihydrothymoquinone; Mansour et al., 2002). In the hepatotoxic model of CCl_4_ treated mice, a single dose of TQ (100 mg/kg) was administered. CCl_4_ induced liver damage along with elevated serum ALT was significantly reversed by TQ. TQ and DHTQ, dose dependently, inhibited non-enzymatic lipid peroxidation induced Fe3^+^-ascorbate in liver homogenate and DHTQ showed a superior action as compared to TQ and BHT (Nagi et al., 1999). In a similar study, acetaminophen induced hepatoxicity in mice were treated with three doses of TQ (0.5, 1, and 2 mg/kg/day) for 5 days orally. A considerable and dose dependent reduction in serum ALT activities, total nitrite/nitrate, lipid peroxides, reduced glutathione, and increase in ATP was observed. The authors conclusively reported the significant resistance of TQ to oxidative and nitrosative stress along with an improvement in the mitochondrial energy production (Nagi et al., 2010).

Renal ischemia is a complex physiological condition characterized by failure of both the kidneys and renal allografts. The efficacy of TQ against renal ischemia/ reperfusion in rat models was fruitfully demonstrated (Bayrak et al., 2008). *N. sativa* oil administration showed pronounced decrease in serum blood urea nitrogen, malonaldehyde, nitric oxide, protein carbonyl content, and creatinine with corresponding an increase in SOD, CAT, GSHPx, and total anti-oxidant capacity. A beneficial effect on hepatic enzymes in streptozotocin-nicotinamide induced diabetic rat models has been reported following treatment with TQ (Pari and Sankaranarayanan, 2009). TQ administered intragastrically at the doses of 20, 40, 80 mg/kg for 45 days dose-dependently improved the heights of insulin and hemoglobin concomitant to reduced glucose and HbA1c. Taken together, the findings of its PPAR-γ agonist and carbohydrate metabolism regulatory property, TQ can be a promising agent for diabetes and diabetic vascular complications. However, the PPAR-γ mediated insulin sensitizing action of TQ yet to be studied.

### Neuroprotective actions of thymoquinone

TQ has been used to treat a variety of neurodegenerative and neuropsychiatric diseases. Hosseinzadeh and Parvardeh showed the efficacy of TQ administered intra-cerebroventricularl in attenuating pentylenetetrazole (PTZ) and maximal electroshock (MES)-induced seizures in rodent models along with effects on pentobarbital induced hypnosis and locomotor activity. TQ administered intraperitoneally at the dose of 40 and 80 mg/kg also found to reduce the onset and duration of myoclonic seizures. Furthermore, TQ impaired the motor co-ordination and reduced locomotor activity inferring that an increase in opioid receptor mediated GABA action attributed its anti-convulsant activity in petitmal epilepsy (Hosseinzadeh and Parvardeh, 2004; Ahmad et al., 2013). In another study, Ilhan et al. established the anti-oxidant and anti-epileptic activity of *N. sativa* oil on PTZ-induced kindling seizures in mice and found superior to sodium valproate, a standard drug for epilepsy. This action was elicited by the potent inhibition of ROS formation by TQ via a variety of mechanisms as described by Hosseinzadeh and Parvardeh (2004) and numerous other studies showing anti-oxidant activities. Ethanol-induced apoptotic neurodegeneration in prenatal rat cortical neurons were treated with metformin and TQ separately and together to investigate the synergistic mechanism. The exposure of 100 mM ethanol for 12 h triggered neuronal death and metformin and TQ both were found to enhance the cell viability and reduce the elevation of cytosolic free calcium as well as normalized mitochondrial transmembrane potential. These effects were attributed to depressed Bcl-2 (anti-apoptotic protein) expression and increase in Bax expression and activation of cytochrome-C. Thus, the alteration of the expression of key proteins such as Bcl-2, Bax, Caspase-9, Caspase-3, and PARP-1 by TQ and Metformin were showed in conferring the therapeutic activities (Ilhan et al., 2005; Al-Majed et al., 2006). Further, Raza et al. (2006) reported that TQ also diminishes sodium valproate induced hepatotoxicity. TQ synergistically enhances the efficacy of sodium valproate at doses 50 and 100 mg/kg in PTZ as well as MES models. TQ co-administered with valproate in drinking water for 21 days, also significantly reduced liver injury marker enzymes in serum such as ALT (alanine transferase) and AST (aspartate transaminase), non-protein sulfhydryl's and attenuated increased lipid peroxidation in hepatocytes. These effects were further studied in a pilot study consisting of 22 children with refractory epilepsy were treated with 1 mg/kg TQ as adjunctive therapy for 4 weeks. The reduction in frequency of seizures was statistically reasoned in the group treated with TQ along with a standard anti-epileptic drug and found that the benefits were up to the parental satisfaction (Raza et al., 2006). Based on these preliminary findings, further studies are necessary to reassure the usage of TQ as adjuvant in epilepsy with contemporary drugs for therapeutic aids as well as minimizing the adverse effects of modern synthetic drugs. Further, TQ also showed attenuation of oxidative stress and attenuated induction of the pro-inflammatory cytokines and elicited neuroprotective effect against epilepsy as well as spinal cord ischemia-reperfusion injury (Gökce et al., 2016).

### Anti-arthritic action of thymoquinone

TQ has been showed effective in rheumatoid arthritis and the protective effects of TQ showed mediated via reduction in interleukins (IL-1β) and tumor necrosis factor (TNF-α) in adjuvant-induced arthritis, the major culprits of inflammatory responses (Tekeoglu et al., 2006; Vaillancourt et al., 2011). Moreover, ovalbumin-induced asthma in mice displayed elevated levels of leukotrienes-B4, C4, Th-2 cytokines, and eosinophils in bronchoalveolar lavage fluid. TQ dealing perfected the pathological perturbations closely associated with airway inflammation by suppressing lipoxygenase (5-LOX) and NF-kβ. El-Gazzar et al. conveyed the initiation of suppressive NF-kβ homodimer obligatory to the promoter in LPS-induced rat basophil cells; RBL-2H3 (El Mezayen et al., 2006; El Gazzar et al., 2007). However, Sethi et al. observed that NF-kβ inhibition is attributed to additional TNF-α-induced Ik-β degradation and phosphorylation along with p65 translocation (Sethi et al., 2008).

Additionally, IL-6 induced STAT3 phosphorylation in U266 multiple myeloma cells were found inhibited by TQ along with c-Src and JAK-2 activation. The study further revealed the interaction of cyclin D1, apoptotic proteins, surviving, Mcl-1 and vascular endothelial growth factor in the U266 cells. TQ interceded decrease in peroxynitrite (${\text{NO}}_{2}^{-}$) remained initiate in parallel with the weakening in iNOS protein manifestation. Further, the anti-inflammatory activity of TQ was evaluated on 96 cytokines. TQ was found to diminish expression of Cxcl10 and different cytokines induced by LPS. TQ was also found to attenuate activated microglia and delay the onset of inflammation-associated neurodegenerative diseases (Kodappully Sivaraman Siveen et al., 2014; Taka et al., 2015).

### Hepatoprotective effects of thymoquinone

In previous years, there are so many reports have been demonstrated the protective effect of TQ on liver toxicity and its consequences. Administration of TQ at the dose of 100 mg/kg by oral gavage in the rats with CCL_4_ induced liver toxicity showed significant improvement form the deleterious effect of CCL_4_. TQ showed strong anti-oxidant property as mentioned in the report that it significantly reduce the malondialdehyde content and increase the levels of different endogenous anti-oxidant s like SOD, CAT, and GSH (Nagi et al., 1999). TQ is also reported to have protective effects in tert-butylhydroperoxide (TBHP)-induced toxicity in isolated rat's hepatocytes. TQ prevented the loss of intracellular enzymes as reduced glutathione and protected the membrane integrity by preventing the further damage of cell membrane as evidenced by reduce the leakage of AST and ALT. Oral administration of TQ showed shielding effect in acetaminophen induced liver toxicity as demonstrated by reduction in the serum activity of amino transferase enzymes. This effect of TQ is might be reducing the generation of reactive oxygen species and nitrosative stress. The TQ had strong ability to enhance the concentration of GST. The previous reports also suggested that, supplementation of TQ increases the quinone reductase and GSH in the liver cells. Also it could beneficial in the induction of the phase-II enzymes in acetaminophen induced liver toxicity in rats (Nagi and Almakki, 2009). B (a) P-treated tumor-bearing mice showed increase in the hepatocyte toxicity as increase in the lipid peroxidation and decrease in the GSH, GST activities. TQ administration showed depletion of the oxidative stress and reactive oxygen species (Badary et al., 1999).

The hepatoprotective efficacy of TQ has been extensively investigated through multiple *in-vivo* experimental models of chemically-induced liver toxicities. In studies involving experimental animals, TQ is reported to protect liver against cadmium (Zafeer et al., 2012), cyclophosphamide (Alenzi et al., 2010), tamoxifen (Suddek, 2014), cypermethrin (Ince et al., 2012), alfatoxin-B1 (Nili-Ahmadabadi et al., 2011), and paracetamol-induced toxicities (Singh et al., 2013). Its hepatoprotective effects against the anti-tubercular drugs induced toxicities in experimental animals is established by Jaswal et al. (2013).

The mechanisms of liver protection conferred by TQ involve its innate anti-oxidative potentials, its ability to induce the anti-oxidative enzymes and, to spare the depletion anti-oxidative mechanisms. In the cadmium-induced liver damage TQ decreases oxidative stress, minimizes the reduction of catalase, and improves the expression of superoxide dismutase (Zafeer et al., 2012). Such evidences of multipronged anti-oxidative efficacies and sparing of the anti-oxidative mechanisms are reported in cases of cyclophosphamide (Alenzi et al., 2010) and tamoxifen-induced liver toxicities in rats (Suddek, 2014). In the hyper-cholesterolemic rats TQ encourages appearance of the liver anti-oxidant genes. It upsurges the expression of SOD, CAT, GSH-P_*X*_ genes and elevates the liver SOD and GSH-Px levels (Ismail et al., 2010). TQ also increases the activities of quinone reductase and glutathione transferase (Nagi and Almakki, 2009). Even in the surgically induced bile duct ligation in rats, TQ inhibits oxidative stress and ductular proliferation (Oguz et al., 2012).

TQ treatment is reported to significantly protect against the toxicity caused by lead (Pb) metal through inhibition of reactive oxygen species generation. In this study, TQ did not reduce the hepatic accumulation of Pb (Mabrouk et al., 2016). It is suggested that TQ possesses a potent and multimodal anti-oxidative potential and makes TQ an important component of the remedies against the liver toxicities induced by drugs and chemical including carcinogens.

Apart from the anti-oxidative effects, TQ is reported to reduce the tamoxifen-induced TNFα levels in serum and liver tissue (Suddek, 2014). In majority of the studies, the hepatoprotective efficacy of TQ has been supported with the evident reductions in the markers of liver damage like AST, ALT, and ALP. Such reductions in the markers of liver damage are reported against the cypermethrin and aflatoxin B1-induced hepatotoxicity in rats (Nili-Ahmadabadi et al., 2011; Ince et al., 2012; Jaswal et al., 2013). A remarkable ability of the TQ to support regeneration of the liver tissue is reported in cypermethrin-induced hepatocellular necrosis in mice (Ince et al., 2012).

Studies on the nanoparticulate formulation of TQ have revealed that TQ inhibited paracetamol-induced liver cirrhosis and fibrosis (Singh et al., 2013). Bai et al. (2014) evaluated molecular mechanisms of protection conferred by TQ against the thiocetamide-induced liver fibrosis. In this study, TQ reversed the liver tissue damage, inflammatory infiltrations and accumulation of the extracellular matrix proteins. TQ found to abridge the mRNA levels of α-smooth muscle actin (α-SMA), collagen-I and tissue inhibitor of metalloproteinase-1 (TIMP-1). It also condensed the countenance of toll like receptor-4 (TLR4) and subsequent rise in the inflammatory cytokine levels. The inhibitory effects of TQ on the phosphatidylinositol 3-kinase (PI3K) phosphorylation along with its stimulatory effects on the phosphorylation of AMPK and liver kinase B-1 specifies that TQ reduces the ECM accumulation through phosphorylation of AMPK signaling pathways.

Apart from its hepatoprotective activity, TQ is also reported to confer protection against the genotoxicity induced by xenobiotics and infectious organisms. Orally administered TQ reduces the N-nitrosodiethylamine (NDEA)-induced hepatic cancer by reducing the liver injury and by down regulating the expression of tumor markers (Raghunandhakumar et al., 2013). In this report TQ is claimed to prevent hepatic nodule formation and reduced the progression of tumor development. TQ exerted anti-proliferative effects and arrested the cell cycle in the G1/S phase (Raghunandhakumar et al., 2013). TQ significantly reduced the chromosomal aberrations induced by schistosomiasis infection in mice. The genoprotective activity of TQ is also evident on the spleen and bone mar-row cells in both *in vitro* and *in vivo* experiments (Aboul-Ela, 2002).

These reports on the hepatoprotective and genoprotective potentials of TQ make it a druggable substance worth of further detailed experimental and clinical investigations as a protectant against hepatic inflammation, fibrosis and carcinogenesis. However, it must be highlighted here that, TQ itself may induce dose-dependent toxicities and hence determination of the dose levels at which it should be explored further.

### Anti-hyperglycemic effects of thymoquinone

Diabetes is a chronic disorder and related to chronic complications for example, problems with the heart functioning, kidney damage, neuropathic pain, and retinopathy. There are so many scientific data available with the traditional or herbal remedies for the diabetes, which makes the development of an alternative therapy for the diabetes and its related complications.

In excess of 1,200 types of plants described for anti-diabetic activities, including *N. sativa* (Marles and Farnsworth, 1995). TQ has anti-diabetic activity and the most useful and clinically relevance model of human diabetes is streptozotocin (STZ)-induced diabetes in experimental animals.

TQ showed strong anti-hyperglycemic activity and reduced the gestational type of diabetes. Administration of TQ for 30 days at the dose of 50 mg/kg to STZ treated hamsters showed significant reduction in blood glucose and glycated hemoglobin. Glucose creation was significantly sunk in hamsters preserved with TQ, it narrowed gluconeogenesis by irresistible the blend of gluconeogenic enzymes (Fararh et al., 2005).

Administration of TQ (6 weeks) reduced STZ-nicotinamide induced diabetes in rats and increases insulin level. TQ improved the utilization of glucose and decreases liver glucose production. TQ (80 mg/kg) reduced the enzymes required for gluconeogenesis (Pari and Sankaranarayanan, 2009). Also it reduced the diabetic state by reducing the level of MDA and glucose. TQ enhanced most of the poisonous properties of STZ, including DNA injury, mitochondrial vacuolization, annihilation, and well-maintained β-cell veracity by lessening superoxide anions. Thus, the anti-diabetic consequence of TQ due to improvement of the cellular and sub-cellular edifices of β cells (Abdelmeguid et al., 2010). Treatment of TQ in STZ preserved rats, congested the countenance of COX-2 enzyme, TBAR levels, along with augmented points of SOD in pancreas (Al Wafai, 2013).

Nutritious supplementation of gestational diabetic mothers through TQ during gestation and lactation ages amended diabetic snags and upheld an effectual T cell resistant reply in their descendants. The uplifting effect of TQ on IL-2 level-cell propagation, and the subsequent liberation of both mingling and thymus homing T cells in the progeny of diabetic mothers heightened the immune retort (Badr et al., 2011). Likewise, it designated a lessening in the fraction of abortions, a growth in the number of actual pregnancies and an upgrading of transience amongst new pups innate to diabetic mothers. TQ better divergent hydro peroxide and oxidative stress in pups and these fallouts may be arbitrated by boom in the levels of GST, GSH, CAT, and a reduction in DNA injury (Kapoor, 2009). Build-up of progressive glycation end-products in tissues and serum, plays important role in diabetes-associated difficulties. TQ had anti-glycation consequence and abridged diabetic malfunctions due to protein glycation (Anwar et al., 2014). It controlled the plasma meditations of cholesterol and triglycerides; plasma triglyceride and cholesterol heights reduced in the TQ treated diabetic rats (Al-Naqeep et al., 2009).

TQ administered orally at the dose of 10 mg/kg ameliorated investigational diabetic neuropathy. TQ showed retrieval of the histopathological changes in sciatic nerves, and myelin break-down diminished evocatively afterward TQ administration (Kanter, 2009). Reactive oxygen species and inflammation play a vital role in the growth of diabetic glitches. STZ-induced diabetes triggered nephropathy, while administration of TQ showed significant improvement in renal morphologic and functional improvement (Kanter, 2009; Pye et al., 2014). TQ was operative for β-cell defense in contradiction of injury, through the decreasing inflammatory activity of NO pathway (El-Mahmoudy et al., 2005).

Intraperitoneal administration of TQ (3 mg/kg) in diabetic rats regularized the raised levels of IL-1β and TNFα (El-Mahmoudy et al., 2005). STZ induces a surge in heart and brain NO and MDA, and reduction in endogenous anti-oxidant s, while employment of TQ improved these levels. Serum levels of creatine kinase-MB and brain types was condensed in the diabetic rats, which enhanced with TQ treatment. In diabetes, there was an obvious strengthening in norepinephrine, dopamine and a noticeable decrease in serotonin level; these remained partially up-turned by TQ administration (Hamdy and Taha, 2009). These verdicts afford systematic base to the widespread use of NS seeds as an anti-diabetic remedy (Al-Hader et al., 1993).

### Effects of thymoquinone in respiratory diseases

The beneficial properties of TQ on respiratory disorders including asthma and dyspnoea have been defined in Iranian ancient medical books (Sharafkandy, 1990). TQ own the ability to neutralize the harmful properties of and injuries caused by chemicals and environmental toxins. TQ weakened lung damage encouraged by long-lasting exposure to toluene in rats mediating the anti-apoptotic mechanisms (Kanter, 2011). It also reduced the progression of pulmonary fibrosis and overexpression of activated NF-kβ in lung tissues induced by bleomycin in rats. TQ corrected emphysema in air alveoli, inflammatory cell infiltration, lymphoid hyperplastic cells initiation near the bronchioles, and reinstated events of anti-oxidant enzymes; SOD and GST (El-Khouly et al., 2012).

TQ was also found effective against cyclophosphamide persuaded pulmonary impairment in rats (El-Khouly et al., 2012). Cyclophosphamide amplified the level of total protein, LDH and TNF-α. TQ treatment for 7 days pre and post cyclophosphamide administration reduced the modifications in lung and serum biomarkers related with inflammation laterally through condensed MDA and refurbishment of anti-oxidant s. It also corrected cyclophosphamide-induced histopathological changes in lung tissues (Suddek et al., 2013). Furthermore, TQ also relaxed pulmonary arterial rings and produced a concentration-dependent decrease in the tautness of the pulmonary arterial rings pre-contracted by phenylephrine. This relaxant consequence was believed to be due to activation of ATP-sensitive K^+^ channels and non-competitive obstruction of serotonin, alpha-1, and endothelin receptors (Suddek, 2010). TQ also showed possible anti-inflammatory activity throughout the allergic response in the lung persuaded by airway challenge of OVA-sensitized mice through the hang-up of Th-2 driven immune response (El Gazzar et al., 2006). Intraperitoneal administration of TQ ameliorated allergic airway inflammation by hampering Th-2 cytokines initiation, cell infiltration and hyperplasia in the airways. TQ reserved IL-4, IL-5, and IL-13, also initiation of IFN-γ production in the BAL fluid (El Gazzar et al., 2006). The attenuation of inflammation by TQ includes hang-up of COX-2 expression and PGD-2 production (El Mezayen et al., 2006). Chronic airway inflammation and leukotrienes are important part of bronchial asthma. Administration of TQ after OVA challenge supressed expression by lung cells of 5-lipoxygenase, the chief enzyme in leukotriene biosynthesis and deteriorated the levels of LTB-4 and LTC-4. This was attended with reduction in Th2 cytokines, BAL fluid and lung tissue eosinophilia (El Gazzar et al., 2006). TQ influenced relaxation of guinea pig's isolated trachea by constraining lipoxygenase products of arachidonic acid metabolism and non-selective delaying of the histamine and serotonin receptors (Al-Majed et al., 2000). Still, TQ has also been shown possible for its use in the action of acute respiratory distress syndrome in rats (Isik et al., 2005). Intra-venous administration of TQ improved intratracheal pressure with minimal effect on the respiratory rate in urethane anesthetized guinea pig (El Tahir et al., 1993). Taken together, the studies demonstrate the therapeutic potential of TQ in respiratory complaints and additional provision the traditional use of black seed used since ancient time to treat respiratory diseases.

### Nephroprotective effects of thymoquinone

The nephroprotective effects of TQ in various pathogenic conditions have been reported. TQ found to ameliorate the appearance of renal lesions resulted from various toxic agents partly by weakening reactive oxygen species and inflammation. TQ treatment ameliorated acute renal failure induced by gentamicin in rats by restoring mitochondrial function and salvaging ATP production. It ameliorated degenerative changes induced by gentamicin and improved the renal function markers such as blood urea nitrogen and creatinine. TQ also inhibited lipid peroxidation concomitant to improved anti-oxidant defense in renal cortex as evidenced by raised GSH level and activities of GSHPx and CAT (Sayed-Ahmed and Nagi, 2007).

TQ prevented mercuric chloride-induced renal damage in rats as evidenced by improved activities of anti-oxidant enzymes and restoration of renal function along with histopathological salvage of renal tissues (Fouda et al., 2008). Administration of TQ in drinking water (50 mg/l) showed to attenuate acute kidney injury induced by cisplatin and improved the therapeutic effects of cisplatin in rodent models. TQ caused reduction in serum urea, creatinine and enhanced polyuria, kidney weight and creatinine clearance (Badary et al., 1997). It also thwarted vancomycin-induced kidney injuries. The anti-biotic, vancomycin produced surge in serum blood urea nitrogen, creatinine, and MDA along with reduced activities of SOD and GSHPx in kidney tissues. TQ treatment corrected vancomycin-induced biochemical changes (Basarslan et al., 2012). TQ also improved renal morphology and produced functional improvement in STZ-induced diabetes in rats (Kanter, 2009).

TQ (5 mg/kg per day) provided in the drinking water earlier and during ifosfamide treatment found to recover the severity caused by ifosfamide. TQ ameliorated ifosfamide prompted phosphaturia, glycosuria, raised serum creatinine, urea and regulated creatinine clearance rate. It also prevented depletion of GSH from renal tissues and diminished lipid peroxide via anti-oxidant action (Badary, 1999). Additionally, TQ also ameliorated doxorubicin (DOX)-induced nephropathy in rats and reduced triglycerides and cholesterol and corrected hyperlipidemia. TQ (10 mg/kg/day) also diminished DOX-induced proteinuria and albuminuria and attenuated total triglycerides, cholesterol and MDA in the kidneys. Besides, non-protein sulfhydryl (NPSH) content and CAT activity in the kidneys of TQ-treated rats obviously raised (Badary et al., 2000). TQ treatment abridged kidney damage by regularizing the raised levels of serum urea, creatinine and urinary albumin excretion in DOX-induced nephrotoxicity. TQ also subdued lipid peroxidation and enhanced the activities of endogenous anti-oxidant s along with reduced level of renal oxidase NOX-4 (Suddek, 2010; Elsherbiny and El-Sherbiny, 2014). TQ showed effective in hepatorenal dysfunction desirous by kidney I/R. kidney I/R caused in a rise in lipid peroxidation and diminution in GST and SOD in liver and kidney tissues, and TQ treatment produced the reverse of these variations. It abridged spermidine/spermine N-1-acetyl-transferase (Awad et al., 2016). Treatment with TQ endangered the kidneys after oxidative stress produced by pyelonephritis. In the pyelonephritis, SOD, CAT activity, and lipid peroxidation levels were abnormal and these were refunded to nearly usual and overturned by TQ treatment in the pyelonephritis (Evirgen et al., 2011). TQ supplementation showed protection in cypermethrin-induced-sloughing off epithelial cell, shrinkage of glomeruli, and necrosis of renal tubules in kidneys of mice (Ince et al., 2012). This suggests that TQ can be promising agent for nephropathies. In our laboratory, we demonstrated the effects of TQ on cyclophosphamide-induced hemorrhagic cystitis in mice. TQ treatment administered intraperitoneally in the doses of 5, 10, and 20 mg/kg before and after the cyclophosphamide injections prevented histological perturbations as evidenced by decreased cellular infiltration, edema, epithelial denudation and hemorrhage in the bladder tissue, reduced oxidative stress, lipid peroxidation and inhibited DNA fragmentation. TQ also induced Nrf2 expression in the bladder tissues of mice concomitant to the restored anti-oxidant s in the bladder tissues (Gore et al., 2016).

### Effects of thymoquinone on reproductive system

*P. aeruginosa* induced bacterial prostatitis with significant increase in the reactive oxygen species. The administration of TQ led to decrease in the oxidative damage and elevated the levels of endogenous anti-oxidant s such as SOD, CAT, and decreases the MDA content (Rifaioglu et al., 2013). TQ also showed the better anti-inflammatory activity by reducing the level of elevated cytokines or pro-inflammatory factors (TNF-α, IL-6, IL-1β) compared to normal one (Inci et al., 2013). Some previous reports suggested the protective effects of TQ by its anti-inflammatory and anti-oxidant effects in cadmium exposure induced testes damage (Fouad and Jresat, 2015). TQ showed protective effects in the lead toxicity induced testicular damage. The lead induces testicular damage by diminishing the spermatogenic and steroidogenic functions. Administration of TQ concurrent with lead improved the functions of testis and testosterone level (Mabrouk and Cheikh, 2014). Administration of TQ weakened interstitial space dilatation in methotrexate induced testis injury in mice; it upturned histological alterations, increased total anti-oxidant enzymes and congested the upsurge in the MPO action (Gökçe et al., 2011).

Moreover, TQ augmented the degree of necrotic cells at attentions amid 2.5 and 20 μM. Also, it produced attention reliant on genotoxicity in hepatocyte main cultures, i.e., an upsurge of the occurrence of chromosomal aberrations and micro nucleated cells. Prominently, TQ had competently slays the cancerous cells deprived of damaging the host cells (Khader et al., 2009; Akhondian et al., 2011). The discerning cell toxicity of TQ for malicious cells associated to usual osteoblasts, mouse renal cells (Kirui et al., 2003), normal human lung fibroblasts, and Vero cells (Shoieb et al., 2003) has been defined. Furthermore, normal cell lines for example primary mouse keratinocytes and Madin-Darby canine kidney (MDCK) cells are described to be resilient to the cytotoxic properties of TQ (IC50 = 101 M; Roepke et al., 2007; Effenberger et al., 2010).

TQ, although depolymerized the microtubule system and boisterous the mitotic spindle connotation of A549 cells, did not interrupt the microtubule system of normal HUVEC cells (Gali-Muhtasib et al., 2008; Acharya et al., 2014). However, straight forwards, TQ might be processed to ROS and rise oxidative stress, which subsidizes to the exhaustion of anti-oxidant enzymes and impairment to DNA in liver cells treated with TQ. TQ caused suicidal erythrocyte death, like apoptotic consequence on nucleated cells. Acquaintance of human erythrocytes to TQ stimulated phosphatidyl serine expose at the red blood cells surface. The properties are predictable to quicken the clearance of red blood cells from circulating blood, therefore encouragement to the development of anemia (Khader et al., 2009). The route of administration of TQ can influence the toxicity. As reported administration of TQ intraperitoneal to rats showed toxicity as pancreatitis. The reason could be intraperitoneal injection caused complete absorption of TQ into systemic circulation. Although, rats with oral gavage administration of TQ showed momentary signs of toxicity, reason might be TQ was metabolized in the liver and degraded as a part in the gastrointestinal tract (Ali and Blunden, 2003).

The dearth of pre-clinical studies through TQ commentary the maximum tolerated dose, the highest quantity is harmless to manage in the absence of unbearable side effects, is stared as restriction in by TQ in clinical locations. Additional investigation together at the clinical and pre-clinical level is therefore wanted to control the therapeutic actual dose of TQ in numerous illnesses. In specific, the consequence of TQ on ionic channels, particularly calcium channels, is still indistinct and wants to be additional exploration.

### *In-Vitro* activities of thymoquinone

Many studies have been performed related to the *in-vitro* activities depending on the various stages of cancer using various cell lines. It also includes the molecular targets involved and the effect of TQ on the molecular targets. Table 3 represent the activities of TQ along with the changes in the molecular targets in particular cell lines.

## Pharmaceutical development of thymoquinone

### Formulations

The universal drawback in the use of phytochemicals for drug development is poor aqueous solubility, non-targeted drug distribution as well as unknown toxicity due to its potent activities. TQ being a phytochemical reported to have such obstacle (Alkharfy et al., 2015). In current years, many methods have been discovered to develop formulations with improved pharmaceutical and pharmacokinetic properties in order to achieve maximum therapeutic benefits. Till date many novel formulations such as carbon nanotubes, polymeric micelles, nanoemulsions, PEGylated/stealth liposomes, dendrimers, solid-lipid nanoparticles, liposomes, and noisome have been developed for TQ.

To overcome the drawback of TQ many researchers can explore the nanotechnology like carbon nanotubes which is effective in preparation of targeted formulation, it can be single walled or multi-walled which causes conjugation of TQ thus enhancing the effect of TQ. Among them, nanotechnology is one of the promising approaches to resolve the above mentioned obstacles by significantly controlling the drug release and localizing the action of drug. Nano formulation (1–100 nm) built approaches can increase the dissolution of poorly soluble drugs in totalling to enlarge their steadiness, bioavailability, and decrease toxicity. Other effective formulations developed are polymeric micelle which help to increase the water solubility of the drug as it entrap the drug in hydrophilic core, PEGylated/stealth liposome prevent the disintegration of the drug that showed increased volume of distribution and residence time of the drug. The dendrimers tend possess both hydrophilic and hydrophobic regions that leads to effective targeting thus it may be an improved formulation for TQ, niosomes are non-ionic surfactant based vesicles and more effective for transdermal route of administration.

## Safety and adverse effects of thymoquinone

There is an upsurge in the use of phytomedicines for their therapeutic and preventive benefits. However, the issue of their safety received enormous attention too before use in humans. There are plenty of literatures on efficacy but the regulatory toxicology studies are lacking to meet the legal requirements in order to encourage for clinical studies and meet the requirements of their use in health and medical care. It has been revealed that the seeds and oil of the plant comprising TQ demonstration very low grade of deadliness or look barren of toxicities (Gökce et al., 2016). Numerous studies were approved to evaluate the toxicological chattels of TQ *in vitro* and *in vivo* (Ali and Blunden, 2003; Abukhader, 2012), and lone an imperfect figure of hearsays on the theoretically poisonous possessions of TQ occurs. The animal models of different diseases where TQ has been studied in different dose ranges have been shown a promising preventive and therapeutic agent, endowed with beneficial effects with negligible toxicities (Al-Shabanah et al., 1998; Mansour et al., 2001; Bai et al., 2014). Recently, administration of TQ (20 days) did not encourage death in Balb/c mice or disturb their mean body weight, an actual subtle limit for toxicity in rodent models (Zafeer et al., 2012). TQ was not found to induce any clinically significant change in neurological function, behavioral parameters, biochemical workroom variables or vigorous ciphers. TQ treated at 1 mg/kg/day was found fine stood (Gali-Muhtasib et al., 2008). Badary et al. showed that TQ (0.03%) supplementary in the drinking water of mice for 3 months did not showed any signs of toxicity, except for a reduction in fasting plasma glucose level. In a study, constant distribution of TQ for 30 days by means of tri-calcium phosphate lysine pill (0.02 g of TQ) to adult male rats have shown negligible adverse effects on the dynamic strictures and no sign of toxicity on reproductive organs (Badary et al., 1997). It is remarkable that the active amounts of TQ were found safe and TQ even in doses of 90 mg/kg/day upon sub chronic administration to rats found devoid of toxicity. The authors also reported hypo activity and difficulty in respiration as signs of toxicity 24 h after TQ administration at high doses (2–3 g/kg). It also reduced GSH content in liver, kidneys, and heart tissues. This additional showed liver and kidney toxicity as established by upsurge in plasma metabolites and enzymes; plasma urea, creatinine, and the enzyme activities of ALT, LDH, and creatine phosphokinase (Ali and Blunden, 2003).

## Concluding remarks and future directions

This review enumerate biological properties of TQ and reveal its therapeutic potential against several diseases including diabetes, neuropathic pain, ulcerative colitis, cancer, cardiac, musculoskeletal, and neurodegenerative illnesses including Alzheimer's and Parkinson's. TQ being a natural constituent in numerous edible plants makes a dietary component since ancient times and considered safe with time tested evidence. The low toxicity of TQ makes it tremendous commercial attention worldwide to be used in foods and gaining acceptance for pharmacological research, therapeutic benefits and pharmaceutical development for human application in coming years. Apart from its medicinal use these days, it has also been used in the food industries like an additive, flavor enhancer and preservative. The available data are suggestive of its use either as nutraceutical or as prophylactic or adjuvant for long-lasting illnesses involving low grade chronic oxidative stress and inflammation. Molecular pharmacology data from several studies depicts that TQ modulates enzymatic, apoptotic, and cell signaling pathway, transcription factors, different receptors and ion channels to elicit its pharmacological effects. The molecular and pharmacological mechanism underlying the therapeutic benefits provide scientific basis of further studies for the pharmaceutical development of TQ. The multifunctional and poly-pharmacological possessions too synergies the action of other drugs and provides a rationale for use in diseases involving multifactorial pathogenesis necessitating one drug many target approach in therapeutics. Its use with other conventional drugs may also improve the scope of its synergistic blend by enhancing effectiveness and diminishing adverse effects. Although the experimental indications are promising, but there is a need to investigate the translational features of TQ. The precise molecular mechanism by which TQ applies its beneficial effects is still poorly understood. Furthermore, the structure-activity relationships of this pharmacophore need to be investigated in detail for development of druggable congeners.

Briefly, the pharmacological properties, favorable pharmacokinetics, relative safety and toxicity, the lipophilicity, high therapeutic index, efficacy and safety profile make TQ a promising candidate for drug development. Notwithstanding the possible excellent advantage of this compound of natural origin, the clinical trials are needed for the translation of the experimental indications into reality in humans.

## Author contributions

All authors contributed to the writing. All authors designed, revised the manuscript, and approved the final version.

### Conflict of interest statement

The authors declare that the research was conducted in the absence of any commercial or financial relationships that could be construed as a potential conflict of interest.

## Abbreviations

TQ: Thymoquinone
SOD: superoxide Dismutase
CAT: catalase
GSH: Glutathione peroxidise
PPAR: Peroxisome proliferator-activated receptors
DTQ: dithymoquinone
SFE 1: supercritical CO2 extract
HD: Hydro-distillation
HPLC: high performance liquid chromatography
Vss: volume of distribution at steady state
COX: Cycloxygenase
PG: Prostaglandin
NADPH: Nicotinamide adenine dinucleotide phosphate-oxidase
DPPH: 1,1-diphenyl-2-picrylhydrazyl
ABTS: 2,2-azinobis 3-ethylbenzothiazoline-6-sulfonic acid
MDA: malondialdehyde
NO: nitric oxide
l-NAME N: omega-nitro-l-arginine methyl esters
NADH: nicotinamide adenine dinucleotide
DHTQ: dihydrothymoquinone
BHT: butylated hydroxytoluene
QR: Quinone reductase
PIP3: Phosphatidylinositol-3,4,5-trisphosphate
STAT3: Signal transducers and activators of transcription 3
DG: diosgenin
PARP, GSK-3β: Glycogen synthase kinase-3
cdk: cyclin-dependent kinase
HO-1: Heme oxygenase-1
NF-kβ: nuclear factor kappa B
TNF: Tumor necrosis factor
GTP: guanosine triphosphate
LPS: Lipopolysaccharides
ERK1/2: extracellular signal-regulated kinase ½
IL-6: Interleukin 6
TQRF: thymoquinone rich fraction
SFE: supercritical fluid extraction
LDLC: low density lipoprotein cholesterol
LDLR: low density lipoprotein receptor
HMG-COAR: 3-hydroxy-3-methylglutaryl- coenzyme A reductase
MICs: minimum inhibitory concentrations
DAPI: 4,6-diamidino-2-phenylindole
GT: glutathione transferase
DHTQ: Dihydrothymoquinone
PTZ: pentylene tetrazole
MES: maximal electroshock
LT-B4: Leukotrienes B4
5-LOX: Lipoxygenase
SLNs: Solid lipid nanoparticles
SMA: smooth muscle actin
TLR4: toll-like receptor 4
LKB-1: liver kinase B
TBARS, NPSH: non-protein sulfhydryl
NOX-4: renal oxidase
SSAT: spermidine/spermine N-1-acetyl-transferase
ABP: Acute bacterial prostatitis
MDCK: Madin-Darby canine kidney
MTD: maximum tolerated dose
TCPL: Tri-Calcium Phosphate Lysine
GTP: guanosine triphosphate.
